# Supplementary figures and images for: Central American and Caribbean tourism destinations’ competitiveness: A temporal approach
Source: PLoS One. 2021 May 25;16(5):e0252139. doi: 10.1371/journal.pone.0252139 (PMC8148355; doi:10.1371/journal.pone.0252139)

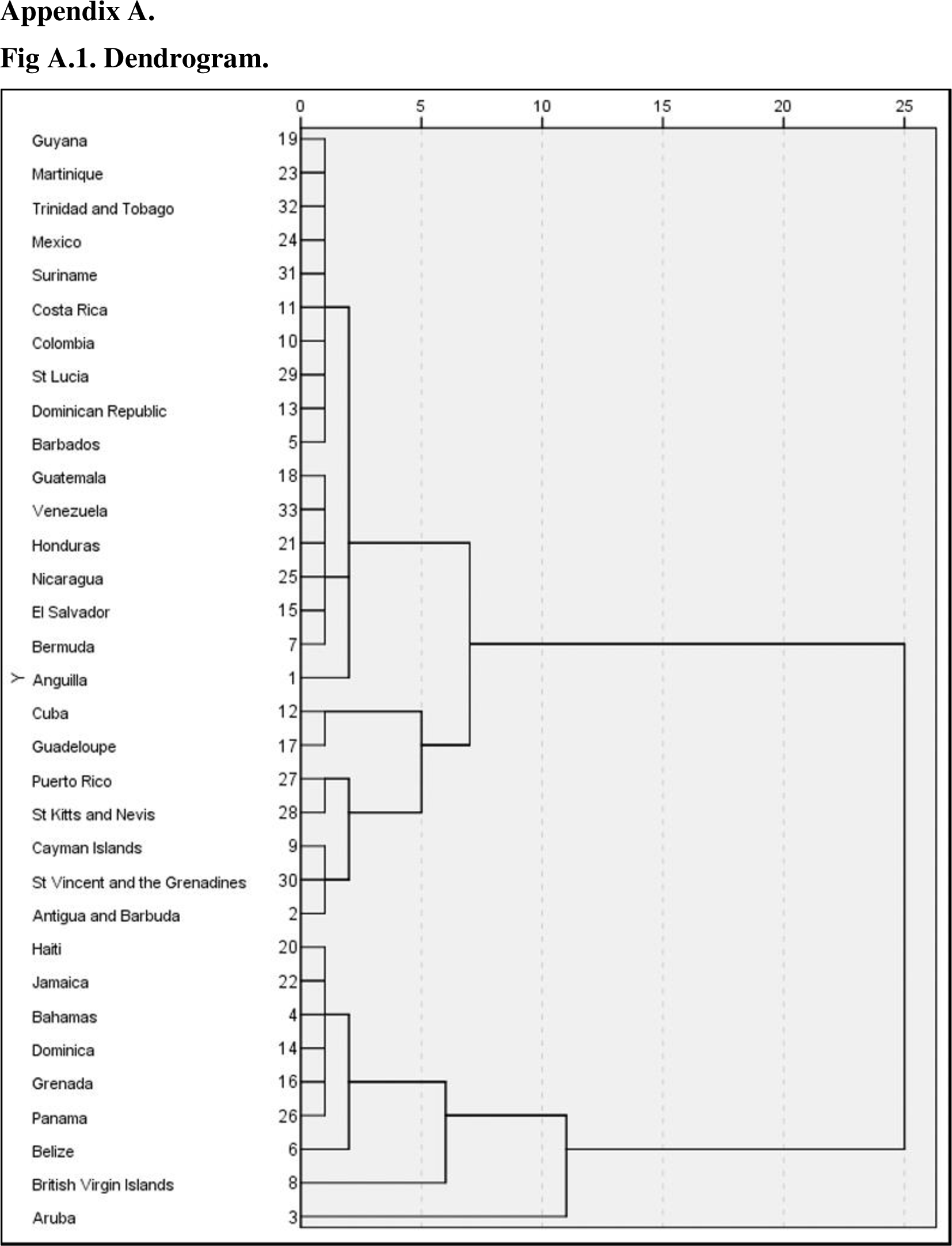

Supplement: S1 Appendix — (TIF) [file pone.0252139.s001.tif]
