# Supplementary material for: Central American and Caribbean tourism destinations’ competitiveness: A temporal approach
Source: PLoS One. 2021 May 25;16(5):e0252139. doi: 10.1371/journal.pone.0252139 (PMC8148355; doi:10.1371/journal.pone.0252139)

PLOS ONE

Central American and Caribbean tourism destination’s competitiveness based on performance. A new approach

--Manuscript Draft--

**Manuscript Number:**	PONE-D-20-32669
**Article Type:**	Research Article
**Full Title:**	Central American and Caribbean tourism destination’s competitiveness based on performance. A new approach
**Short Title:**	Tourism destination’s competitiveness based on performance
**Corresponding Author:**	Victor Ernesto Pérez León, PhD<br>Universidad de Sevilla Facultad de Ciencias Económicas y Empresariales<br>Sevilla, Andalucía SPAIN
**Keywords:**	Tourism destination competitiveness; Linear Regression; Cluster Analysis; Performance; Central America and The Caribbean
**Abstract:**	This study presents a new method for measuring tourism destination competitiveness based on their performance. Issues such as type of data gathered, tools and methods employed, destinations size and their number are addressed. The proposal is based on the information given by linear regression equations, which allows observing destination behavior over time and facilitates their comparison. The data used was from the period 2000-2019. Cluster Analysis was introduced for grouping destinations according to their performance. A comparison among destinations was carried out and using the non-aggregative and an aggregative approach. Some destinations attained a better performance than others considered as more competitive in global international rankings. Five clusters were clearly identified. The results were consistent with the World Travel and Tourism Council outputs.
**Order of Authors:**	Victor Ernesto Pérez León, PhD
	Maria Amparo León Sánchez
	Flor Mª Guerrero
**Additional Information:**	
**Question**	**Response**
**Financial Disclosure**<br><br>Enter a financial disclosure statement that describes the sources of funding for the work included in this submission. Review the submission guidelines for detailed requirements. View published research articles from *PLOS ONE* for specific examples.<br><br>This statement is required for submission and **will appear in the published article** if the submission is accepted. Please make sure it is accurate.	L. M. A. Grant Number: PI-195, University of Pinar Del Río, Cuba https://www.upr.edu.cu<br><br>P. V. E. Grant Numbers: P18-RT-1566, UMA18-FEDERJA-065, Agencia de Innovación y Desarrollo de Andalucía<br><br>F. G. Grant Number: SEJ332. Secretaría General de Universidades, Investigación y Tecnología, Junta de Andalucía

Powered by Editorial Manager® and ProduXion Manager® from Aries Systems Corporation

**Unfunded studies**<br>Enter: *The author(s) received no specific funding for this work.*<br><br>**Funded studies**<br>Enter a statement with the following details:<br>• Initials of the authors who received each award<br>• Grant numbers awarded to each author<br>• The full name of each funder<br>• URL of each funder website<br>• Did the sponsors or funders play any role in the study design, data collection and analysis, decision to publish, or preparation of the manuscript?<br>• **NO** - Include this sentence at the end of your statement: *The funders had no role in study design, data collection and analysis, decision to publish, or preparation of the manuscript.*<br>• **YES** - Specify the role(s) played.<br><br>* typeset	
**Competing Interests**<br><br>Use the instructions below to enter a competing interest statement for this submission. On behalf of all authors, disclose any competing interests that could be perceived to bias this work—acknowledging all financial support and any other relevant financial or non-financial competing interests.<br><br>This statement **will appear in the published article** if the submission is accepted. Please make sure it is accurate. View published research articles from *PLOS ONE* for specific examples.	The authors have declared that no competing interests exist.

**NO authors have competing interests**<br><br>Enter: *The authors have declared that no competing interests exist.*<br><br>**Authors with competing interests**<br><br>Enter competing interest details beginning with this statement:<br><br>*I have read the journal's policy and the authors of this manuscript have the following competing interests: [insert competing interests here]*<br><br>* typeset	
**Ethics Statement**<br><br>Enter an ethics statement for this submission. This statement is required if the study involved:<br><br>• Human participants<br>• Human specimens or tissue<br>• Vertebrate animals or cephalopods<br>• Vertebrate embryos or tissues<br>• Field research<br><br>Write "N/A" if the submission does not require an ethics statement.<br><br>General guidance is provided below. Consult the submission guidelines for detailed instructions. **Make sure that all information entered here is included in the Methods section of the manuscript.**	N/A

**Format for specific study types**<br><br>**Human Subject Research (involving human participants and/or tissue)**<br>• Give the name of the institutional review board or ethics committee that approved the study<br>• Include the approval number and/or a statement indicating approval of this research<br>• Indicate the form of consent obtained (written/oral) or the reason that consent was not obtained (e.g. the data were analyzed anonymously)<br><br>**Animal Research (involving vertebrate animals, embryos or tissues)**<br>• Provide the name of the Institutional Animal Care and Use Committee (IACUC) or other relevant ethics board that reviewed the study protocol, and indicate whether they approved this research or granted a formal waiver of ethical approval<br>• Include an approval number if one was obtained<br>• If the study involved *non-human primates*, add *additional details* about animal welfare and steps taken to ameliorate suffering<br>• If anesthesia, euthanasia, or any kind of animal sacrifice is part of the study, include briefly which substances and/or methods were applied<br><br>**Field Research**<br><br>Include the following details if this study involves the collection of plant, animal, or other materials from a natural setting:<br>• Field permit number<br>• Name of the institution or relevant body that granted permission	
**Data Availability**<br><br>Authors are required to make all data underlying the findings described fully available, without restriction, and from the time of publication. PLOS allows rare exceptions to address legal and ethical concerns. See the PLOS Data Policy and FAQ for detailed information.	Yes - all data are fully available without restriction

A Data Availability Statement describing where the data can be found is required at submission. Your answers to this question constitute the Data Availability Statement and **will be published in the article**, if accepted.<br><br>**Important:** Stating 'data available on request from the author' is not sufficient. If your data are only available upon request, select 'No' for the first question and explain your exceptional situation in the text box.<br><br>Do the authors confirm that all data underlying the findings described in their manuscript are fully available without restriction?	
**Describe where the data may be found in full sentences. If you are copying our sample text, replace any instances of XXX with the appropriate details.**<br><br>• If the data are **held or will be held in a public repository**, include URLs, accession numbers or DOIs. If this information will only be available after acceptance, indicate this by ticking the box below. For example: *All XXX files are available from the XXX database (accession number(s) XXX, XXX.).*<br>• If the data are all contained **within the manuscript and/or Supporting Information files**, enter the following: *All relevant data are within the manuscript and its Supporting Information files.*<br>• If neither of these applies but you are able to provide **details of access elsewhere**, with or without limitations, please do so. For example:<br><br>*Data cannot be shared publicly because of [XXX]. Data are available from the XXX Institutional Data Access / Ethics Committee (contact via XXX) for researchers who meet the criteria for access to confidential data.*<br><br>*The data underlying the results presented in the study are available from (include the name of the third party*	The data used in the research is available in the DATAGATEWAY of the World Travel and Tourism Council https://wttc.org/Research/Economic-Impact/Data-Gateway

and contact information or URL).<br>• This text is appropriate if the data are owned by a third party and authors do not have permission to share the data.<br><br>* typeset	
Additional data availability information:	Tick here if your circumstances are not covered by the questions above and you need the journal’s help to make your data available.

Powered by Editorial Manager® and ProduXion Manager® from Aries Systems Corporation

Manuscript Click here to access/download;Manuscript;Manuscript.docx

Central American and Caribbean tourism destination's competitiveness based on performance. A new approach

Víctor Ernesto Pérez León[1,3*], Maria A. León Sánchez[2], Flor Guerrero[3]

[1] Department of Applied Economics II, Faculty of Economics and Business Sciences, University of Seville. Seville, Spain.

[2] Mathematics Department, Faculty of Technical Sciences, University of Pinar del Río. Pinar del Río, Cuba.

[3] Economics, Quantitative Methods and Economic History Department, Faculty of Business Sciences, Pablo de Olavide University. Seville, Spain.

[*] Corresponding Author:

E-mail: vpleon@us.es

1. Introduction

Several countries are improving their tourism sector to contribute to their economic and social development. This is why the amount of tourism destinations is increasing worldwide. At the same time, the quantity of originating markets has augmented, but to a lesser degree. This asymmetry has led to fierce competition in the international tourism market, which is constantly on the rise (1). This perspective justifies the allocation of public resources to attract more visitors to destinations by increasing their competitive position in relation to others (2). As a result, tourism destination competitiveness (TDC) has emerged as an important concept for policymakers and scholars and there is an extensive debate regarding the definitions, parameters and measurement of the concept (3).

TDC is a topic that is complex and not well understood (3) and it still has no commonly accepted and standardized definition (4). There is an absence of consensus regarding just what destination competitiveness means (5). In this sense, the view that has a greater practical acceptance is derived from the analysis of performance in the sector (tourist arrivals, tourism receipts, etc.), as well as from its similarity to the flows of goods (exports), despite this appeal being less theoretical (6, 7). Performance refers to the evolution of the tourist sector outputs that can be seen in physical (e.g., number of tourists), monetary (e.g., tourism receipts), or qualitative (e.g., average stay or average expenditure per overnight spent at the destination) terms (7), which are useful to evaluate a destination's competitiveness. Additionally, it should be noted that measuring and comparing tourism competitiveness is not an easy task (8). Therefore, several authors have created or adopted different models for measuring tourism competitiveness. Among the myriad of tools used to analyze competitiveness are

Porter's five forces model (9), surveys that capture the consumer's perceived image of the destination (10) and models based on price competitiveness (11).

Furthermore, as is stated in the bibliography, different studies have been developed for measuring tourism competitiveness for a single destination (12, 13), or for various (6, 14-16). Others consider a given moment (14, 17-24) or a time span (7, 25-28). Moreover, the analysis concerning methodology, number of destinations and their size still remains.

Notwithstanding, the literature has not yet arrived at a consensus on the most appropriate measure of competitiveness (28). There are some articles focused on the analysis of performance (6, 7, 25) but these are rather scarce. Performance refers to the evolution of the tourist sector (7). Consequently, it is possible to assume that a higher-than-average rate for the indicators analyzed could be considered a gain of competitiveness (29).

In this sense, this study aims to analyze the performance of tourist destinations toward a better competitive position during a time span. The proposal involves observing a destination's performance with respect to its competitors (17, 24, 30), and also with respect to itself (1, 31). To attain this goal, it is proposed to use the slope of the regression equation for each indicator in each destination. This allows identifying the average performance of a destination in a time span. As a result, competitiveness can be analyzed as a dynamic and not as a static approach.

The proposal allows including all information available in each indicator, in such a way that a destination's performance is not only affected by the initial and final values, but by all the intermediate values from the time span. Additionally, Cluster Analysis is proposed to group destinations according to their performance level in the indicators studied. This study involves destinations from Central America and the Caribbean due

to the low presence of these countries in important international rankings such as the Travel and Tourism Competitiveness Index (TTCI) of the World Economic Forum (WEF), despite the importance of this economic activity for most of the countries of the region. Only 17 of them have been included in the global ranking and four were omitted from the 2017 edition due to data unavailability. Besides, in spite of the broad research on tourism destination competitiveness measurement, the literature has still not arrived at a consensus on its most appropriate measure (28).

For the purpose of most existing destination competitiveness studies, however, a tourism destination is conceptualized as a defined geographical region, which is understood by its visitors as a unique entity, with a political and legislative framework for tourism marketing and planning (32). This research involves 33 tourism destinations between Island States and Continental States from Central America and the Caribbean. The selection of the competitors responds to the criteria of geographical proximity and market share. These countries compete for attracting tourism mostly from the USA and Europe. That makes them potential competitors, typically in cruise tourism, which is strong in the region.

The data used for the study correspond to the variables registered by the World Travel & Tourism Council (WTTC) for all destinations. It comprises 12 indicators representative of the tourism industry of the destinations. This study is one of the largest TDC research works carried out in the area so far, since it jointly considers a great number of indicators and destinations (6, 25, 26, 28, 33, 34).

2. Tourism destination competitiveness measurement

One of the key issues in tourism competitiveness is how to measure it. Abreu-Novais, Ruhanen (3) have excellently developed this topic. The authors argue that measuring tourism competitiveness involves four aspects: The type of data gathered, the tools and

methods employed, the level of destination used and the number of destinations chosen for the comparison. The data used can be based on either hard data (objective measures) or soft data (subjective values). As pointed out by these authors, classic hard data is typically included in assessments of destination competitiveness (6, 26, 35, 36).

There are other studies that use soft data, or stakeholders' perceptions in which TDC is based on the valuation of different topics (1, 17, 37, 38). One major advantage of this data is its ability to capture the intrinsic characteristics of destinations, which are important factors in competitiveness and are normally difficult to measure. The combination of both types of data is the most desirable way to measure TDC. In addition, this possibility is only achieved by a few initiatives, like the Travel and Tourism Competitiveness Index (TTCI) of the World Economic Forum (WEF) (23, 24).

In spite of the advantages of subjective measures, sometimes its use for comparing destinations according to their competitiveness level may be not completely feasible. Mostly when the destinations under evaluation are regions or countries, it may occur that not all the respondents have complete knowledge about competitiveness topics in a given destination or in all the destinations compared. Moreover, when the study involves destinations within the same region, the use of questionnaires as a tool to collect their data can compromise response rates (3, 39). Such is the case of the WEF's Travel and Tourism Competitiveness Index, in which each respondent of the Executive Survey Opinion offers a score for an indicator considering his/her own country and not with respect to its competitors.

In large studies, it is very difficult to achieve the use of respondents with information about all the destinations considered. This does not mean that the use of perceptions to analyze TDC is a mistake; on the contrary, their use helps to value the TDC determinants that are qualitative, multidimensional, abstract or imprecise, e.g.: culture,

history and perceived quality, among others. The usefulness of judgments in TDC measurement is justified by the fact that this kind of measures has progressively dominated this stream of research (3, 40, 41). Taking this into account, the authors would simply like to point out an issue that needs to be considered when using subjective data. This is to ensure that all the respondents have full knowledge of the aspects analyzed in each destination compared.

The second matter concerning TDC measurement refers to methods and tools used. There are several methodologies registered in the literature, each one having its strengths and weaknesses. They are chosen according to the researcher's intentions and study objectives. Principal Component Analysis (PCA) is one of the most utilized, for its ability to reduce information (17, 42) as well as Factor Analysis (43) and Cluster Analysis (9, 44). Novel Multi-Criteria Decision Analysis approaches can be found (45) and other applications, such as Multiple Attribute Utility Theory (MAUT) (30), TOPSIS (46), ELECTRE (16) and AHP (47). Also, Data Envelopment Analysis is widely used (15, 48) as is Importance Performance Analysis (IPA), which has recently gained popularity in the field (3) and is used in diverse studies (10, 31). In general, it should be stated that there is no methodology signaled as the one most preferred to measure TDC. The choice of methodology involves the decision maker's preferences and depends on its facility to analyze the results obtained.

Destinations' size is the third topic concerning tourism competitiveness studies. It has been addressed at different levels: resorts (9, 49), tour operator and hotel companies (48); cities (50), municipalities (17); regions (30) and countries (5, 15). Goffi and Cucculelli (17) present a detailed list of applications for a diverse size of destinations.

The number of destinations is the fourth signaled subject. This depends on the study range, the stakeholders' necessities and which places are considered to be the

destination's competitors. There are studies focused on a single destination (1, 41) in which the researchers want to determine if a destination is competitive or not according to the respondents' considerations and/or indicator values, or to test a new TDC model (51). This quantity may increase up to three (35, 52), ten (53) or more (54). The amount can be higher (6, 17, 23, 24) and depends on the researcher's intention. Studies with a wider number of destinations are mostly associated with TDC rankings (24, 28). Furthermore, the number of destinations can by conditioned by information availability. For instance, in TDC studies, while most developed countries do collect reliable tourism data, less developed countries struggle to provide accurate and timely statistics (55) and these tend to be fewer or to be not considered.

Additionally, the authors would like to highlight a subject related to the time span for which the TDC is analyzed. Most studies use data from a single moment. This implies obtaining a unique measure of each indicator (9, 15, 20, 23, 42, 44, 56, 57). In these studies, TDC is viewed as a static phenomenon because its value represents the destination's state at a specific moment. This approach is useful to evaluate destinations and compare them with respect to the others. However, it cannot be used to analyze a destination's performance over time, unless the same measurement is carried out with the same set of indicators at another moment.

Meanwhile others, fewer, use data from a period (6, 26, 48) and incorporate measures for the same indicators in a time span (26), in such a way that it is not the direct value of the indicators that is analyzed, but their performance over a period. Performance refers to the evolution of the tourist sector (7) and performances of destinations have been influenced by their competitiveness (10, 31). Consequently, it is possible to assume that a higher-than-average rate for the indicators analyzed could be considered a gain of

competitiveness (29). Indicator growth rates are approximate to the concept of competitiveness because they indicate the change of those levels acquired over time (7). A destination’s performance can be observed through a single value and a unique calculus operation is required and not as many as the intervals comprised in the period. In terms of TDC, it is important for policy makers to evaluate if a destination performs better than its core competitors do at a given moment. Moreover, it will be appreciable if a destination’s self-improvement or failure rate over time can be identified. In such a way, TDC is viewed as a dynamic phenomenon, consistent with its nature.

Considering that for most TDC studies, the competitiveness level of a destination is associated with the way in which it achieves high scores for the indicators observed, this research aims to analyze the way in which a destination improves indicator values toward a better competitiveness positio[illegible]o attain this goal, destinations from Central America and the Caribbean will be studied based on the information of a group of indicators for the period 2000-2019 and their improvement or decreasing rate determined in such a way that all the available information will be taken into account. A destination’s performance will be based on the way it achieves better indicator values over time with respect to itself and its competitors.

Considering the key topics for measuring TDC, this study uses completely objective data. Despite the usefulness and dominance of subjective indicators in TDC studies, hard data helps to conveniently gather large volumes of data and destinations (3) and leads to more precise and accurate results (20). What is more, the exclusion of subjective indicators eliminates the need of external information and the search for experts or stakeholders with complete knowledge of the competitiveness issues considered for all the destinations.

The proposed methodology is based on linear regression equations. These are easy to obtain and comprehensible. Furthermore, linear regression is available in most statistical software and considers all values for every indicator in each year included; that is why there is no information lost. The value obtained considers all the data. Outcomes are affected positively or negatively for good or bad indicator values respectively, which is a desirable characteristic in measurement processes. The results are realistic and represent the average rate at which a given destination improves or declines in a specific indicator over the period considered. As a result, competitiveness is viewed as a dynamic process because the scores are single values which are representative of a destination's performance during a period.

The number of destinations and their sizes are not problems for the proposal. First, all the destinations considered as competitors may be included. The scores allow ranking them according to their own behavior in each indicator. In the same line, the sizes are not an issue because the measurement process demonstrates the destination's behavior in each indicator with respect to itself in a time span. The mainstream TDC studies assume that one destination is more competitive than another if it obtains a better value for most of the indicators measured, which is a real assumption. This proposal also implies that a given destination could be considered more competitive if it is able to improve its indicators' values with respect to itself more than others do over time.

3. Tourism in the region

63.36% (21) of the countries involved are Island States and 12 (36.64%) are Continental States. All of them, except Puerto Rico, are Associated with or Members of the Caribbean States Association (CSA); 22 belong to the Caribbean Tourism Organization (CTO) and, according to the World Tourism Organization (UNWTO) classification, all belong to the Caribbean or Central American Region, except Mexico (North America)

and Guyana, Suriname and Venezuela (South America). For the purpose of this study, all these countries will be grouped into the same Region, considering the competitive destination approach exposed by Abreu-Novais, Ruhanen (3). Other small islands and continental states of the area were not considered because of the lack of information. In general, for most of these countries, tourism is their main source of income.

The 33 countries included in the study account for 33% and 36% of the international visitors to the continent between 2000 and 2019, acting as a second region within the American Continent. According to the World Tourism Organization information (UNWTO), this region has maintained a growing tendency during the period. It was less affected by the decline of the number of visitors in 2009. As a result, it is possible to assume that this group of countries had a higher tourist affluence in the time registered as the most critical during the period analyzed. In addition, starting from 2013, this group of countries had a tendency to increase more noticeably than North America, in spite of having a lower number of tourists. This confirms that the growth of the number of visitors in the Americas is due to the countries under analysis. Despite the augmentation of international tourist arrivals to the Continent, this sub-region achieved the highest increment ratio.

The United States remained the most important tourist supplier to the region with a growth of 6.3% increase in arrivals. Approximately 14 million Americans visited the region, with most arriving in Mexico, the Dominican Republic, Jamaica, Puerto Rico and the Bahamas. Canadian arrivals showed an increased growth of 4.5%, arrivals from Europe rose by 4.3% and Intra-regional travel as a whole grew by 11.4% (58).

With regard to the possibilities of tourist emissions of the area, a short analysis allows appreciating that Caribbean inhabitants practically do not frequent the zone as tourists. For Mexico and the Central American countries, Caribbean visitors do not reach 1%,

while for South American countries they represent 3.91%. Generally, tourists from the Caribbean prefer or are more likely to be tourists in their area, rather than in the rest of the continent. A glance at the main tourism receptors worldwide reveals that the presence of Caribbean tourists is small, which shows the few travel possibilities that the inhabitants of the region have and justifies their greater presence as tourists in their own territory. In addition, this highlights the weaknesses in terms of economic development that they present with respect to the rest, which contributes to explaining their scarce presence among global tourism providers.

The 46.7 million international visitors who visited the region in 2016 spent US$31.4 billion which meant a total of $56.4 billion in GDP and 2.4 million jobs. Meanwhile, the domestic market generates more than 25% of the region's Travel and Tourism GDP. Overall, tourism contributes 15.2% of the Caribbean's GDP and 13.8% of its employment. However, in many Caribbean countries, the sector accounts for over 25% of their GDP – more than double the world average of 10.4% (59). In addition, starting from 2013 the region has had an increasing trend which is more noticeable than in North America, in spite of having a lower number of tourists. This confirms that the growth of the number of visitors in the Americas is due to the countries under analysis. Despite the augmentation of international tourist arrivals to the Continent, the Caribbean achieved the highest increment ratio according to the World Tourism Organization's information (60, 61).

According to the WTTC information, 11 countries from the region were recorded among the top 35 most tourist-dependent worldwide (28). In 2016, three more countries of the area were included within this sample of 35, measured by the contribution of the tourism industry to their GDP. Furthermore, 11 of these countries are once or twice among the top ten worldwide in some of the issues measured by the WTTC. This

representativeness has been maintained for the last ten years (26, 28), which is a demonstration of the dependence of this region on tourism.

4. Methodology

4.1 Data

Taking into account that there is no single set of indicators applicable to all destinations at all times, the data used for the study correspond to the values registered by the World Travel and Tourism Council (WTTC) All the indicators fulfill the requirements for desirable tourism data: availability, accessibility, reliability, timeliness, comparability and accuracy (27). The success of the tourism destination development depends on both the supply side and the demand side. Tourism destination competitiveness on the demand side is closely related to the quality of the whole tourism experience. On the supply side, destination competitiveness is more concerned with the economic benefits of the destination (revenues, employment, the sustainable growth of the destination and the firms within this destination) (62). Therefore, the joint use of indicators that are representative of both demand and supply is convenient to analyze TDC.

Moreover, research that analyzes TDC in a time span use few indicators (usually no more than five) (7, 25-28). However, this study involves indicators used by the WTTC to evaluate the result of the tourist activity in each destination through the contribution of tourism to GDP, plus the Direct Spending Impacts and the Indirect and Induced Impacts (63). To all this is added to the difficulty of obtaining reliable values for the indicators in developing countries, as is recognized in the literature (25-28).

Information was available for all destinations, considering that, from the list of 33 countries in the study, less than 16 are included in all the editions of the WEF's Travel and Tourism Competitiveness Index (TTCI), this being mainly caused by the lack of information. What is more, four of these destinations were excluded from the editions of

2017 and 2019 due to insufficient data (24, 64). The indicators selected correspond to hard data. This allows for a broader number of destinations in the study. Soft data was not included. Firstly, because its use is more related to the analysis of a single destination. Secondly, due to the inexistence of persons with partial or absolute knowledge of tourist issues for all the destinations considered. The indicators used in the study are listed below. Their values are calculated by the WTTC and presented in their reports in current money, US$ billion and percentage.

1. (GDP_DC) Travel & Tourism Direct Contribution to Gross Domestic Product: GDP generated by industries that deal directly with tourists, including hotels, travel agents, airlines and other passenger transportation services, as well as the activities of restaurant and leisure industries that deal directly with tourists.
2. (GDP_TC) Travel & Tourism Total Contribution to Gross Domestic Product: GDP generated by direct Travel & Tourism industries plus the indirect and induced contributions, including the contribution of capital investment spending.
3. (EDC) Travel & Tourism Direct Contribution to Employment: The number of direct jobs within the Travel & Tourism industries. This includes employment by hotels, travel agents, airlines and other passenger transportation services (excluding commuter services). It also includes, for example, the activities of the restaurant and leisure industries directly supported by tourists.
4. (ETC) Travel & Tourism Total Contribution to Employment: The number of jobs generated directly in the Travel & Tourism industry plus the indirect and induced contributions.
5. (DTTS) Domestic Travel & Tourism Spending: Spending within a country by that country's residents for both business and leisure trips. Multi-use consumer durables are not included since they are not purchased solely for tourism purposes.

6. (LTTS) Leisure Travel & Tourism Spending: Spending on leisure travel within a country by residents and international visitors.
7. (BTTS) Business Travel & Tourism Spending: Spending on business travel within a country by residents and international visitors.
8. (VE) Visitor Exports: Spending within the country by international tourists for both business and leisure trips, including transportation spending.
9. (ITTC) Internal Travel & Tourism Consumption: Total revenue generated within a country by industries that deal directly with tourists including visitor exports, domestic spending and government individual spending. This does not include spending abroad by residents.
10. (GI) Government Individual Travel & Tourism Spending: Government spending on individual non-market services for which beneficiaries can be separately identified. These social transfers are directly comparable to consumer spending and, in certain cases, may represent public provision of consumer services. For example, it includes the provision of national parks and museums.
11. (CI) Capital Investment: Capital investment spending by all sectors directly involved in the Travel & Tourism industry. This also constitutes investment spending by other industries on specific tourism assets, such as new visitor accommodation, passenger transportation equipment, as well as restaurants and leisure facilities for specific tourism use.
12. (OTS) Outbound Expenditure: Spending outside the country by residents on all trips abroad. This expenditure occurs almost exclusively by resident visitors outside the economic territory or on trips to leave this economic territory (using a non-resident carrier for instance.

As is expressed by the WTTC in each country's Economy Impact, the percentage of the total refers to each indicator's share of the relevant whole economy indicator such as the GDP for indicators 1, 2, 5, 6, 7, 9 and 12. For indicators 3 and 4, the percentage is with respect to the whole economy's employment. The percentage of Visitor Exports (8) is relative to the total exports of goods and services. Government Individual Travel & Tourism Spending (10) is relative to the total tourism expenditure. Finally, Capital Investment (11) is relative to the whole economy's investment. These values are available in the WTTC DATA GATEWAY (https://www.wttc.org/economic-impact/country-analysis/data-gateway) and in each country report. The percentage was the one used in the study to avoid data scale differences. The fact that not all the indicators are evaluated with respect to the destinations' GDP is a desirable characteristic. This avoids the possibility of an increase in the contribution of tourism to the GDP of any indicator being caused by the decline of other activities and not because of a higher development of a tourist destination.

4.2 Linear Regression Model

The proposed approach to evaluate TDC is Linear Regression Modeling. This has been previously used in tourism studies for forecasting tourism demand (65). Its main objective is to determine the degree to which one or more variables (independent) affect the dependent variable and has diverse advantages (66). The proposed methodology involves estimating the linear regression equation of each indicator for each destination. The indicators are the dependent variables, measured in percentages, while the independent variable is the time (in years). All the dependent variables are expressed in a 0-100 scale and therefore a normalization process is not required. The intercept represents the indicators' average value at time zero; that is, at the beginning of the period. The slopes of linear regression equations represent the annual performance mean

rate of each indicator in the period with respect to the reference economic value, as is stated in the explanation of each indicator.

The use of these values as initial information enables the researchers to study the destination's behavior in a period without the need to repeat the calculus for each year. It is possible to compare destinations considering their self-performance in each indicator over time. Furthermore, slope values allow comparing destinations according to their average growth rate because of their unit invariance. This method has been proposed due to the possibility of analyzing a destination's behavior taking into consideration all the values of each indicator in the period. That is why all the available information is considered. A variation rate between two periods (initial and final) could be used but this would only consider the initial and final values, avoiding the effect of the intermediate values throughout the time span.

The approach permits observing the behavior of a destination by means of a single value for each indicator in a given time span. This is another way to observe a destination's competitiveness. A destination may improve its competitiveness level over time if it achieves a growth rate in its indicators' values. The main objective is not to forecast, but to observe the average performance of the destinations in each indicator. This is the main advantage of the procedure applied with respect to the others. Moreover, the slope is used as a unique measurement unit for all the destinations to determine groups with a similar growth rate in their indicators. The use of other methods could provide better fits but, in some cases, different for each relationship. The use of different methods may cause an incomparability between destinations.

A negative value for the slope for the ith indicator of the jth destination indicates a negative linear association. This means that the indicator's value decreases over time at a rate equivalent to the slope (Fig. 1, a). Otherwise, a positive score demonstrates an

annual improvement ratio equivalent to the slope (Fig. 1, b). There are other issues, such as the absence of association and other strong relations, but not linear ones. For two given destinations (*i* and *k*) evaluated in a certain indicator *θ*. If $\beta_{0i} > \beta_{0k}$, this denotes that destination *i* has a higher initial expected value than destination *k* at the time 0; that is, in the initial year. If for the whole period, $\beta_{1k} > \beta_{1i}$, this indicates that destination k has a better average growth rate than destination i. As a result, if both destinations maintain the same slope (β_1), destination *k* is going to achieve a higher value than *i* for the indicator and $\theta_k > \theta_i$ as the variable x increases, which means as time goes by.

< Insert Figure 1>

4.3 Cluster Analysis

The use of cluster analysis is proposed to view how destinations are clustering according to their behavior in the period and to identify common patterns. This approach has been used in tourism studies because of its ease in identifying a group of units with similar characteristics according to the phenomena measured (67, 68). This is a multivariate method with the primary purpose of grouping. It is a very common statistical technique in which a set of objects (e.g., events, people, etc.) is subdivided into groups (clusters) in such a way that objects in the same group are more similar (based on certain variables) to each other than to those belonging to other groups (68).

A hierarchical cluster analysis with the Ward's method criterion was applied. This method was used for its ability to minimize differences within clusters and avoid problems generated by the chain. All the variables in it intervene to determine the distance between clusters. Besides, the sum of squares within the cluster is minimized in each step of the clustering process. (69). The square Euclidean distance was used as a

measurement, as is suggested in the specialized bibliography when the Ward method is used (69).

A hierarchical method was applied because non-hierarchical procedures have several disadvantages. Firstly, they require that, in principle, the number of groupings must be inferred, which is an undesirable aspect, since the study wishes to investigate the groups that are formed from the average annual rate of each destination in each indicator. Secondly, the choice of the grouping seed highly influences the procedure. Furthermore, if seeds are chosen by the statistical package, their choice depends on the computer reading order. In this way, it is possible to obtain different outputs with similar data sets. Lastly, the procedure is frequently unfeasible from the calculation point of view because there are too many possible choices, not only due to the number of groups but because of the seed's location (70). It is common to find applications in which the arithmetic mean or the median is used to compare clusters with variables with repeated measures over time. However, the slope is considered as a better option to measure the average behavior in data with a tendency.

5. Results and Discussion

5.1. Non aggregative approach

Perhaps not all destinations follow a linear trend in each indicator because of tourist flows. However, due to the necessity of establishing a comparison, it is not possible to use the model that best matches the behavior of each destination in each separate indicator, but in the one that is applicable to all the model and which facilitates an explanation of the results. With this aim, linear regression could be considered one of most certain approaches due to its characteristics. The results are presented in Table 1, where the slopes of the regression equations of each indicator for each destination are shown.

Table 1. Destinations' slopes

Destinations	GDP_DC	GDP_TC	EDC	ETC	DTTC	LTTS	BTTS	VE	ITTC	GI	CI	OTTS	**Rank**
Anguilla	0.122	0.235	-0.246	-0.757	0.081	0.137	-0.018	-0.758	0.246	0.042	-0.156	-0.074	**28**
Antigua & Barbuda	-0.271	-0.526	-0.271	-0.526	0.056	-0.267	-0.005	-0.559	-0.465	0.110	1.219	0.057	**32**
Aruba	0.752	2.507	0.755	2.248	0.165	0.696	0.052	1.471	0.589	0.086	0.764	0.218	**1**
Bahamas	0.157	0.386	-0.238	-0.125	0.040	0.169	-0.015	0.472	0.075	-0.004	0.597	-0.021	**16**
Barbados	-0.035	-0.152	-0.092	-0.315	-0.006	-0.050	0.015	-0.016	-0.066	0.041	0.427	-0.151	**25**
Belize	0.362	1.157	0.314	1.037	0.042	0.322	0.038	0.736	0.374	0.051	1.107	-0.132	**13**
Bermuda	-0.013	0.247	-0.064	0.156	0.230	-0.023	0.010	-0.285	0.106	0.024	0.187	-0.138	**19**
British Virgin Islands	0.462	1.234	-0.615	-1.271	0.042	0.450	0.013	1.037	0.578	0.101	0.354	0.302	**27**
Cayman Islands	-0.156	-0.377	-0.180	-0.458	0.041	-0.165	0.007	-0.569	-0.226	0.108	0.398	0.237	**30**
Colombia	-0.017	-0.051	-0.015	-0.040	-0.032	-0.006	-0.011	0.241	-0.003	0.003	-0.116	0.013	**14**
Costa Rica	-0.113	-0.215	-0.055	-0.131	0.009	-0.088	-0.027	-0.264	-0.118	0.020	-0.022	-0.089	**26**
Cuba	-0.060	-0.143	-0.046	-0.116	-0.006	-0.062	0.002	-1.516	-0.128	0.020	0.842	-0.037	**29**
Dominican Republic	0.219	0.673	0.202	0.614	-0.031	0.187	0.030	0.741	0.362	0.015	0.205	-0.023	**8**
Dominica	-0.038	-0.096	-0.071	-0.164	0.029	-0.042	0.003	0.254	-0.028	0.074	0.084	-0.033	**17**
El Salvador	0.080	0.219	0.077	0.207	0.096	0.062	0.017	0.288	0.118	0.011	0.238	-0.023	**6**
Grenada	0.221	0.713	0.218	0.665	-0.016	0.085	0.135	0.647	0.414	0.040	0.702	-0.046	**9**
Guadeloupe	-0.069	-0.301	-0.038	-0.250	-0.013	-0.054	-0.012	-1.998	-0.069	0.004	-0.204	-0.140	**33**
Guatemala	-0.003	0.035	0.000	0.037	-0.004	0.001	-0.004	0.007	-0.015	0.018	0.376	-0.009	**7**
Guyana	0.000	-0.012	0.004	-0.008	0.010	0.019	-0.020	-0.174	-0.054	0.027	-0.352	0.011	**22**
Haiti	0.085	0.250	0.058	0.184	-0.015	0.072	0.013	0.833	0.104	0.004	0.264	0.053	**5**
Honduras	0.057	0.216	0.050	0.189	0.075	0.021	0.036	-0.021	0.032	0.015	0.332	-0.055	**10**
Jamaica	0.093	0.394	0.078	0.339	0.081	0.039	0.054	0.838	0.154	0.047	0.461	0.056	**2**
Martinique	0.019	0.021	0.027	0.031	-0.023	0.032	-0.014	-0.239	0.009	0.005	-0.200	-0.057	**20**

Mexico	-0.010	-0.018	-0.113	-0.201	-0.041	-0.012	0.001	-0.053	-0.074	0.030	0.067	0.013	**18**
Nicaragua	0.131	0.298	0.031	0.150	0.069	0.104	0.026	0.036	0.119	0.007	0.198	0.014	**3**
Panama	0.193	0.513	0.250	0.578	-0.011	0.202	-0.009	1.144	0.303	0.018	-0.094	-0.018	**12**
Puerto Rico	0.010	0.026	-0.004	-0.003	-0.010	0.011	-0.001	0.013	0.014	0.020	1.093	-0.070	**11**
St. Kitts & Nevis	-0.066	0.271	-0.058	0.243	0.008	-0.224	0.155	-0.265	-0.214	0.039	0.960	0.095	**24**
St. Lucia	-0.025	-0.131	-0.025	-0.131	0.069	-0.076	0.049	0.125	0.045	0.028	-0.209	0.016	**21**
St. Vincent & the Grenadines	-0.297	-0.603	-0.236	-0.495	-0.064	-0.247	-0.053	-0.256	-0.523	0.032	0.493	-0.006	**31**
Suriname	-0.024	-0.093	-0.040	-0.125	-0.022	-0.014	-0.010	-0.168	-0.036	0.006	-0.011	-0.136	**23**
Trinidad & Tobago	-0.012	-0.019	-0.037	0.023	0.150	0.019	-0.031	-0.129	0.029	-0.007	-0.031	-0.072	**15**
Venezuela	0.022	0.075	0.005	0.021	0.049	0.034	-0.012	0.088	0.018	0.007	0.152	0.043	**4**

The data were not transformed for their use in linear regression. The indicators, valued in percentages, were used to avoid scale differences and eliminate variable transformation. If data transformation had been necessary, then it would have affected the explanatory power of the score used. What is more, the remaining scales in which the data was offered in the WTTC (e.g., the local currency and US$ billion in Nominal and Real prices) do not contribute to the destination's differentiation, due to value similarity caused by data approximation. From an initial set of 7926 values resulting of 12 matrices of order 20 x 33, now we have only 396. These values are the slopes of the regression equations of each destination in each indicator for the period 2000 - 2019.

These values represent the annual performance for each destination in each indicator during the period 2000-2019 and comprises the information of the values for the complete time span. As a result, the average annual performance is affected for all the years included. Despite the important reduction of the information, the study must be developed under the non-aggregative approach. The slopes act as the initial information for each destination.

An analysis of the extreme marks shows that the highest values for all the indicators are in only five destinations. These countries achieved the highest annual increase of all the issues analyzed in the period. Aruba has the best achievement in seven indicators. The two related to employment, (EDC) and (ETC), also those representatives of the contribution to the GDP (Direct and Total), the tourism spending within the country (VE), the ITTC and LTTS. The next is Antigua and Barbuda, with higher yearly growth rates in the two indicators that represent the efforts of the government and the sectors related to tourism in the development of the sector: Government Individual Travel & Tourism Spending (GI) and Capital investment (CI). The last are Bermuda, with the best increment in Domestic Travel & Tourism Spending (DTTS), the British Virgin

Islands, with the best slope for the Outbound Tourism (OTS) and Saint Kitts and Nevis, with the best improvement in business trips in the region (BTTS).

All the worst performances are negative and distributed among seven destinations. Saint Vincent and the Grenadines has the highest decline in five aspects: (BTTS), (GDP_DC), (GDP_TC), (ITTC) and (DTS). This seems to be the less competitive destination or, at least, the one that more average worseness registered in the period, in number of indicators. The British Virgin Islands has the worst performance in (EDC) and (ETC). Then, Antigua and Barbuda, Barbados, Guadeloupe, Guyana and Trinidad and Tobago, achieved the worst performance in (LTTS), (OTS), (VE), (CI) and (GI) respectively.

A detailed inspection reveals only three destinations which have improved in all the indicators for the period: Aruba, Jamaica and Nicaragua. Despite the behavior of these destinations in all the indicators considered, there exist other destinations with values higher than those in their indicators. This means that these destinations have augmented their scores at a higher rate than the others across the period. Consequently, in comparison to the others, they have achieved a better competitiveness position with respect to themselves. As a result, a positive performance in all the indicators on their own is not enough; a higher value is also necessary to be considered the most competitive. Belize and El Salvador have an increased level in all indicators except one (OTS), while Haiti has the same behavior but, the negative values was attained in the (DTTS).

From the indicators' point of view, it is possible to note the importance given to tourism in all the countries in the area. A general local government concern about the development and maintenance of non-market services for which beneficiaries can be local and international tourists in the region is evident because of the 31 positive values of the 33 possible for slopes of Governmental Individual Travel & Tourism Spending.

Only Bahamas and Trinidad and Tobago obtained a negative value, very close to zero. In a direct relationship to that explanation above, a common regional interest is observed in the development of travel and tourism services. This is justified by the 23 countries of the sample with positive values for their slopes in Capital Investment. It is evidence of the importance given to this economic activity in the area. This is consistent with the relative best position of the region in this matter (71).

On the contrary, despite the positive performance in GI and CI in the region, and the presence of more average yearly growth than decline, the most widespread local difficulties are related to the capacity to create jobs in the tourism industry. The direct and total contribution to employment shows a decreasing behavior in 19 and 18 of the 33 economies, respectively. This should be a general concern for these countries with the aim to improving the local population`s life conditions. In spite of this almost generalized negative performance, this was the region for which the Travel and Tourism industry most contributed to the total employment in 2017 (in relative terms), according to the WTTC (2018b).

Furthermore, in agreement with the WTTC's forecast, this area is expected to achieve the highest relative growth in this indicator for 2028 (71). However, the most concerning indicator is the one related to the tourism emission of the region. This is consistent with the condition of these destinations as underdeveloped countries. A glance at the main tourism receptors worldwide reveals that the presence of Caribbean tourists is small, which shows the few travel possibilities that the inhabitants of the region. In addition, this highlights the weaknesses in terms of economic development that they present with respect to the rest, which contributes to explaining their scarce presence among global tourism providers.

An analysis between islands and continental states can also be made. Both groups have similar performances in GI and CI, consistent with the regional interest in supporting tourist services and products in favor of tourism development. Additionally, for most countries of both groups, 57.14% of island states and the 58.33% of continental states, the total contribution of tourism to GDP (GDP_TC) has had an annual growth in the period. This is more intense for island states. This issue situates these economies in a more tourism-dependent condition, while continental countries have sources, others than tourism, for supporting their economic development.

The higher difference between these groups is due to the Total Contribution of tourism to the employment with a yearly increment for 28.6% of islands versus 66.7% of continental states. Moreover, island states have a year average growth higher than the continental states in the investment in new visitor accommodation, passenger transportation equipment, as well as restaurants and leisure facilities for specific tourism use (76.2% vs. 58.3%).

Mexico could be viewed as the most competitive destination of the sample. It is included among the top ten destinations worldwide for almost all the indicators measured, except for VE, BTTS and CI. This is consistent with this country’s size and its great and recognized tourist offer, valid to be located within the best according to the WTTC every year. However, this proposal observes how much a destination has been able to improve the topics analyzed with respect to itself in a period. As a result, Mexico is far from being the one with the higher average growth rate, with only four indicators with positive behavior along time, from 2000 to 2019. This means that, despite being one of the most competitive worldwide, this destination does not present a positive performance along the period. However, the values of its indicators are so large in comparison with the destinations in the area that, even maintaining a decreasing rate,

their absolute values are higher than the remaining destinations. As a consequence, our proposal allows us to observe how other destinations in the region have been able to increase the values of their indicators over time in a greater way than Mexico, but with respect to themselves.

4.1 Results for Cluster Analysis

Cluster analysis will allow looking for similarities in TDC performance in the region's destinations. The results clearly identified five groups. The number of groups was decided starting from the dendrogram information (Fig A.1, Appendix A) and the result of the F tests, which reveal great differences among groups, as well as the Kruskal-Wallis test. Additionally, the researchers' criteria for explain group characteristics. The first two are formed by a single destination: cluster 1, Aruba; cluster 2, the British Virgin Islands. The remaining three clusters contain 7, 7 and 17 destinations respectively. These appear in Figure 2, from C1 (first cluster) to C5 (fifth cluster).

< Insert Figure 2 >

The Kruskal-Wallis test demonstrates that 8 of the 12 variables considered cause significance difference among groups (Table 2). Four variables do not contribute to the group's differentiation. Government Travel & Tourism Spending (GI), for which most countries of the region achieved a positive behavior in the period. The other two variables are Business Travel & Tourism Spending (BTTS) and Domestic Travel & Tourism Spending (DTTS), with more than the 50% of the destinations with positive behavior, but a small difference between maximum and minimum values. Furthermore, the region is located in one of the last positions worldwide for these two items according to the WTTC (2018b). Moreover, the prevalence of negative values for the Outbound Expenditure of the region. The Kruskal-Wallis test results and the WTTC outputs were consistent.

Table 2. Kruskal Wallis Test

	Test Statistics[a.b]											
	BTTS	LTTS	E_TC	GDP_TC	ITTC	DTTS	GDP_DC	GI	CI	VE	E_DC	OTTS
Chi-Square	4.339	18.424	11.414	16.002	18.899	3.908	18.357	7.696	12.637	21.685	11.595	6.681
df	4	4	4	4	4	4	4	4	4	4	4	4
AsympSig.	.362	.001	.022	.003	.001	.419	.001	.103	.013	.000	.021	.154

a. Kruskal Wallis Test

b. Grouping Variable: Number of initial cases

The first two clusters, each formed by a single destination (Aruba and the British Virgin Islands). Aruba was the destination with highest slopes in seven indicators. These are comprised among the eight d the sample that causes significance differences among the clusters. Furthermore, Aruba attained the second highest value in other indicator (DTTS) and does not account negative values. The British Virgin Islands has the highest value only in Outbound tourism (OTS); however, accounts the second highest values in four more indicators which causes significant differences. Moreover, this destination registered the highest negative behavior in the two indicators related to the contribution of tourism to the employment. These two countries are located among the top ten destinations worldwide with best achievement levels in GDP_DC, GDP_TC, VE, LTTS and CI according to the WTTC.

Figures 3, 4 and 5 demonstrate the behavior of the slopes for each destination in each indicator in the period. The third cluster comprises five island states and two continental states (Fig. 3). In general, these destinations have an average positive performance in almost all the indicators, with the exception of the outbound expenditure, which does not contribute significance difference among groups. The seven destinations maintained a growing rate in LTTS, GDP_DC, GDP_TC, ITTC and VE. These variables are all significant in showing differences among groups. Moreover, in other four indicators, a single destination attained a negative value, which demonstrate the overall positive behavior of this cluster. Among these indicators can be found those concerning to job creation (E_DC, E_TC) and those related to the effort mad by governments and individuals in favor of tourism development (GI and CI). This cluster can be associated with general good tourism development due to values higher than the mean of the region for almost all the indicators under consideration, with the exception of BTTS, DTTS, GI, CI and OTS.

< Insert Figure 3 >

The fourth cluster is comprised of seven countries (Figure 4), all island states. This cluster demonstrates a global negative behavior. Their positive performance was attained in the GI and CI, as in the previous cluster, consistent with the general behavior of the region. However, with the exception of these two indicators, four or more destinations decreasing rates in all the indicators. This cluster can be associated with general bad tourism development. The values of the indicator considered have been worsening in average along the period and the most concerning issue is related to the number of jobs created by the tourism sector. Guadeloupe and Saint Vincent and the Grenadines only accounts one and two positive slopes respectively. The destinations of this cluster have an average score higher than the mean of the sample for the GI and OTS.

< Insert Figure 4 >

The fifth cluster is the biggest, with 17 countries (Figure 5), from which the majority are continental destinations (10). There are only seven island states. This cluster apparently does not have as homogeneous a behavior as the others, where the changes in the variables are in the same direction. For the third and fourth clusters (Figures 3 and 5) most of the slopes are below or above zero, respectively.

< Insert Figure 5 >

A detailed inspection of Figure 5 reveals that 99% of the slopes are between -0.36 and 0.38, despite the seemingly erratic behaviors. On a more reduced scale, these values would be observed around zero. This is the cluster that registered the average smaller difference between the maximum and minimum slopes. The number of positive and negative slopes were similar for almost all the indicators with the exception of the GI with a single negative value, consistent to the regional view and only the 35% of the

slopes positive, also similar to the overall behavior for Outbound Expenditure (OTS). The average performance for the countries of the cluster is lower than for the region. They only have an average score higher than the mean of the sample for the DTTS. As could be noticed, despite the reduction of the information, it is not possible to attain a ranking for the destinations.

5.3 Aggregative Approach

The analysis was made using the non-aggregative approach. With the aim to obtain a ranking, four composite measures are proposed. The first is called PositSlope and is obtained as the number of indicators for which destinations achieved positive slopes. In the case of tie, then, the composite measurement would be the sum of the positive values. If the tie still remains, then, the values of the negative slopes will be subtracted to the sum of the positive.

The second proposal is the Mean of the slopes for each destination, and the third composite index is the Median. These are descriptive values easy to obtain. The Mean is a compensatory methodology because it admits the compensation between positive and negative scores in such a way that negative behaviors affect the destinations' scores comprises all the indicators, which is a good characteristic for composite indexes. However, the method is influenced by extreme values. The Median is a most robust measure because it is not influenced by extreme values. Notwithstanding, it only considers the values located in the middle of the distribution.

The fourth method is the Restrictive indicator. It is calculated as the sum of the values for the positive slopes of a destination, if it has no negative values. In the case of the presence of negative slopes, then, the value of the Restrictive indicator will be the sum of these negative values. This is a non-compensatory method, because do not admits the presence of both, positive and negative slopes in a destination.

Table 3. Pearson Correlation

	Posit_Slope	Mean	Median	Restrictive
PositSlope	1	.924**	.871**	.766**
Mean		1	.941**	.825**
Median			1	.772**
Restrictive				1
**. Correlation is significant at the 0.01 level (2-tailed).				

The Pearson's correlation coefficient (Table 3) was calculated for these values and the Spearman Rho for the rankings (Table 4). Each pair of methods attained coefficients values statistically significant at 0.01. As a result, it is possible to affirm that the rankings are statistically similar. The ranking corresponding to the restrictive method (Restrictive) appears in the last column of Table 1 (Rank).

Table 4. Spearman's rho Correlation

		Posit_Slope	Mean	Median	Restrictive
Spearman's rho	PositSlope	1	.862**	.899**	.783**
	Mean		1	.933**	.790**
	Median			1	.713**
	Restrictive				1
**. Correlation is significant at the 0.01 level (2-tailed).					

A pair comparison of the rankings reveals that the higher number of destinations that maintain the same positions are located the PositSlope and the Restrictive rankings. However, they have an average variation of 4.42 units, due to the wide variation of the British Virgin Islands and Colombia (17 and 15 positions respectively). The British Virgin Islands worsens in the Restrictive index compared to the PositivSlope. This is because, despite having just two negative slopes, these are the higher for these two indicators in the sample. Therefore, this destination attained a low composite value with the Restrictive approach. On the other hand, Colombia improved 15 positions with the Restrictive method. This result is because, even though this destination has only three indicators with positive behavior in the period, the sum of the negative behaviors is lower than for other destinations, even those with a smaller number of negative slopes, like the case of the British Virgin Islands, for example. As a consequence, it is

important for the destinations to have no negative performances or, at least, to guarantee small values for the negative slopes.

6. Conclusions

The proposed method allows considering a high number of destinations and years. It is easy to develop, the results are comprehensible and demonstrate another way to analyze TDC, based on the slope of the regression equation for the indicator and the destination.Their values indicate the behavior, positive or negative, for each destination in a given period and are not influenced by their size or turism development level. Tourism competitiveness was addressed according to the issues signaled by Abreu-Novais, Ruhanen and Arcodia (2016).

Destinations were defined as whole countries for this study because of the higher probability of obtaining accurate values for the indicators. Hard data or objective indicators were used because of their availability and the impossibility of obtaining subjective values for all the destinations considered in each year of the period. Moreover, the authors considered it very difficult to find a reliable source of subjective information concerning all the destinations included in the study. Its novelty consists in the use of the slopes of the regression equation calculated for each destination and indicator as being representative of the destination's performance in the period. The research has shed light on the way to analyze TDC.

The advantage of the study was the possibility of comparing a destination's performance in a given period without the need to develop calculations for each individual year. Higher and lower slope values were located, generally, in small island destinations with less participation in global rankings publications because of incomplete information. Besides, the best behavior corresponds to destinations with a lower level of economic development. The study also allowed comparing destinations

without taking into account their size, or amount, or stages of their tourist products. As a result, this method is applicable to all the destinations without bearing in mind their size. A destination's improvement level in a period was used to determine its ability to attain a best competitiveness position. The slope of the regression equation for each indicator/destination is representative of the destination's performance over time. Cluster analysis revealed five clearly recognizable groups. Moreover, it was possible to identify a common pattern in the region through the indicators signaled by the Kruskal-Wallis Test, such as those that do not cause a difference of significance among groups.

As a result, it is possible to affirm that there is a general concern for tourism development in the region, supported by the common interest of local governments to contribute to it. This is assumed because of the annual increase in Governmental Travel & Tourism Spending for 32 countries. Furthermore, there is the importance given to the creation of tourist infrastructures and the support of services directly related to tourism development with results for Capital Investment. Additionally, the spending on domestic trips within the same territory has discreetly augmented, but less than in other regions, consistent with the WTTC (2018b). There is a similar behavior in trips for business purposes to the countries of the area, which is lower with respect to other geographical regions and the negative behavior of the Outbound Expenditure, due to the economic conditions of the region

Island states have a better achievement in Business Travel and Tourism Spending, Internal Travel and Tourism Consumption and Capital Investment. The performance of Islands in this last indicator is caused by their higher dependence on this activity compared to the other development possibilities for continental countries. Meanwhile, Continental States have experienced a growing tendency with respect to themselves higher than Islands in the number of jobs generated by tourism, both, Direct ant Total.

For the remaining indicators, the presence of positive behaviors was similar for the number of Islands and Continental States. In general, it was possible to analyze the behavior of one of the most tourist-intense regions worldwide with a detailed analysis of its countries. The research contributes to the paradox of TDC and to the wide group of initiatives to analyze it. The study respects the relative nature of the TDC concept because the scores involve indicator information during the whole-time span analyzed. What is more, slope values explained the degree to which each destination annually improved or worsened in each indicator.

The results were consistent with the latest publications of the WTTC, for which the Caribbean region was first in issues concerning TDC, in relative terms, worldwide (71, 72). Moreover, the results allowed identifying the countries that most influenced this global behavior in the area. In general, this research proposes another way to analyze TDC and it was possible to prove that measuring destination competitiveness is not an easy task. The analysis was carried out by both, non-aggregative and aggregative approaches.

For this final aim, four aggregation methods were proposed to create global rankings. All the proposals have their pros and cons. However, all were valid to attain close results. Considering this, future researches should be based on the creation of a more robust composite indicators, considering the methodological approaches and steps cited in the literature (55). This research results important for decision makers to determine the aspects that must be addressed to improve its competitiveness, giving them the chance of having a more detailed visualization of the most influential factors, positively or negatively, of the destination's performance and, therefore, of supporting the development of initiatives or policies that improve the tourist development. Moreover, a

higher number of indicators and years could be included in the process to increase the time span analyzed.

Acknowledgements

Authors would like to acknowledge the scholarship from the Asociación Universitaria Iberoamericana de Postgrado (AUIP).

References

1. Drakulić Kovačević N, Kovačević L, Stankov U, Dragićević V, Miletić A. Applying destination competitiveness model to strategic tourism development of small destinations: The case of South Banat district. Journal of Destination Marketing & Management. 2018;8:114-24.
2. Webster C, Ivanov S. Transforming competitiveness into economic benefits: Does tourism stimulate economic growth in more competitive destinations? Tourism Management. 2014;40:137-40.
3. Abreu-Novais M, Ruhanen L, Arcodia C. Destination competitiveness: what we know, what we know but shouldn't and what we don't know but should. Current Issues in Tourism. 2016;19(6):492-512.
4. Hong WC. Competitiveness in the Tourism Sector: A Comprehensive Approach from Economic and Management Points. Amsterdam: Physica-Verlag; 2008.
5. Knežević L, Dwyer L, Matjaž K, Mihalič T. Drivers of Destination Competitiveness in Tourism: A Global Investigation. Journal of Travel Research. 2016;55(8):1041-50. doi:10.177/0047287515617299.
6. Bolaky B. Tourism competitiveness in The Caribbean. CEPAL Review August 2011. 2011:55-76.
7. de la Peña MR, Núñez-Serrano JA, Turrión J, Velázquez FJ. A New Tool for the Analysis of the International Competitiveness of Tourist Destinations Based on Performance. Journal of Travel Research. 2019;58(2):207-23.
8. Bãlan D, Balaure V, Veghes C. Travel and tourism competitiveness of the world's top destinations: An exploratory assessment. Annales Universitatis Apulensis Oeconomica. 2009;11:979-87.
9. Claver-Cortés E, Molina-Azorín JF, Pereira-Moliner J. Competitiveness in mass tourism. Annals of Tourism Research. 2007;34(3):727-45.
10. Enright MJ, Newton J. Tourism destination competitiveness: a quantitative approach. Tourism Management. 2004;25(6):777-88.
11. Dwyer L, Forsyth P, Rao P. The price competitiveness of travel and tourism: a comparison of 19 destinations. Tourism Management. 2000;21(1):9-22.
12. Hossain MK, Islam S. An Analysis of Destination Attributes to Enhance Tourism Competitiveness in Bangladesh. African Journal of Hospitality, Tourism and Leisure. 2019;8(2):1-17.

13. Miller M, Henthorne TL, George BP. The Competitiveness of the Cuban Tourism Industry in the Twenty-First Century: A Strategic Re-Evaluation. Journal of Travel Research. 2008;46:268-78.

14. Hanafiah Mohd H, Zulkifly Muhammad I. Tourism destination competitiveness and tourism performance: A secondary data approach. Competitiveness Review: An International Business Journal. 2019;29(5):592-621.

15. Wu WW, Lan L, Lee Y-T. Critiquing the World Econnomic Forum's concept of destination competitiveness: A further analysis. Tourism Management Perspectives. 2012;4:198-206.

16. Botti L, Peypoch N. Multi-criteria ELECTRE method and destination competitiveness. Tourism Management Perspectives. 2013;6:108-13.

17. Goffi G, Cucculelli M. Explaining tourism competitiveness in small and medium destinations: the Italian case. Current Issues in Tourism. 2018:1-31.

18. Gómez-Vega M, J Picazo-Tadeo A. Ranking world tourist destinations with a composite indicator of competitiveness: To weigh or not to weigh? Tourism Management. 2019;72:281-91.

19. Pérez León VE, Pérez F, Contreras Rubio I, Guerrero FM. An approach to the travel and tourism competitiveness index in the Caribbean region. International Journal of Tourism Research. 2020;n/a(n/a).

20. Pulido-Fernández JI, Rodríguez-Díaz B. Reinterpreting the World Economic Forum's global tourism competitiveness index. Tourism Management Perspectives. 2016;20:131-40.

21. Rodríguez-Díaz B, Pulido-Fernández JI. Analysis of the Worth of the Weights in a new Travel and Tourism Competitiveness Index. Journal of Travel Research. 2020;0(0):0047287519899982.

22. Salinas Fernández JA, Serdeira Azevedo P, Martín Martín JM, Rodríguez Martín JA. Determinants of tourism destination competitiveness in the countries most visited by international tourists: Proposal of a synthetic index. Tourism Management Perspectives. 2020;33:100582.

23. WEF. The Travel & Tourism Competitiveness Report 2015. Growth through Shocks. Geneva, Switzerland: World Economic Forum; 2015. 503 p.

24. WEF. The Travel & Tourism Competitiveness Report 2017. Paving the way for a more sustainable and inclusive future. Geneva, Switzerland: World Economic Forum; 2017. 365 p.

25. Craigwell R. Research Paper No. 2007/19. Tourism Competitiveness in Small Island Developing States. Helsinki, Finland: UNU World Institute for Development Economics Research (UNU-WIDER); 2007.

26. Croes R. Measuring and explaining competitiveness in the context of small island destinations. Journal of Travel Research. 2011;50(4):431-42.

27. Croes R, Kubickova M. From potential to ability to compete: Towards a performance-based tourism competitiveness index. Journal of Destination Marketing & Management. 2013;2:146-54.

28. Jackman M, Lorde T, Lowe S, Alleyne A. Evalulating tourism competitiveness of small island developing states: a revealed comparatie advantage. Anatolia: An International Journal of Tourism and Hospitality Research. 2011;22(3):350-60.

29. Dupeyras A, MacCallum N. Indicators for Measuring Competitiveness in Tourism: A Guidance Document: OECD Publishing; 2013.

30. Cracolici MF, Nijkamp P. The attractiveness and competitiveness of tourist destinations: A study of Southern Italian regions. Tourism Management. 2008;30:336-44.

31. Dwyer L, Dragićević V, Armenski T, Mihalič T, Knežević Cvelbar L. Achieving destination competitiveness: an importance–performance analysis of Serbia. Current Issues in Tourism. 2016;19(13):1309-36.

32. Buhalis D. Marketing the competitive destination of the future. Tourism Management, 21(1). 2000:97-116.

33. Craigwell R, Worrell D. The competitiveness of selected Caribbean tourism markets. Social and Economic Studies. 2008;57(1):72-107.

34. De Keyser R, Vanhove N. The competitive situation of tourism in the Caribbean area methodological approach. The Tourist Review. 1994;49(3):19-22.

35. Li G, Song H, Cao Z, Wu DC. How competitive is Hong Kong against its competitors? An econometric study. Tourism Management. 2013;36(1):247–56.

36. Zhang H, Gu C-l, Gu L-w, Zhang Y. The evaluation of tourism destination competitiveness by TOPSIS & information entropy - A case in the Yangtze River Delta of China. Tourism Management. 2011;32(2):443-51.

37. Armenski T, Marković V, Davidović N, Jovanović T. Integrated Model of Destination Competitiveness. Geographica Pannonica. 2011;15(2):58-69.

38. Evren S, Kozak N. Competitive positioning of winter tourism destinations: A comparative analysis of demand and supply sides perspectives–Cases from Turkey. Journal of Destination Marketing & Management. 2018;9:247-57.

39. Dillman DA, Sinclair MDS, Clark JR. Effects of questionnaire length, respondent-friendly design, and a difficult question on response rates for occupant-addressed census mail surveys. Public Opinion Quarterly. 1993;57(3):289-304.

40. Ganguli S, Husain A. A qualitative analysis of Singapore's medical tourism competitiveness. Tourism Management Perspectives. 2017;21:74-84.

41. Komppula R. The role of individual entrepreneurs in the development of competitiveness for a rural tourism destination - A case study. Tourism Management. 2014;40:361-71.

42. Shousheng C, Chunfeng L, Teng G. Research on Tourism Competitiveness of Chinese Island Counties: Based on Factor and Cluster Analysis. Chinese Journal of Population Resources and Environment. 2012;10(4):30-4.

43. Dwyer L, Mellor R, Livaic Z, Edwards D, Kim C. Attributes of destination competitiveness: a factor analysis. Tourism Analysis. 2004;9(1-2):91-101.

44. Kayar ÇH, Kozak N. Measuring destination competitiveness: An application of the travel and tourism competitiveness index (2007). Journal of Hospitality Marketing & Management. 2010;19(3):203–16.

45. Carayannis E, Ferreira F, Bento P, Ferreira J, Jalali M, Fernandes B. Developing a socio-technical evaluation index for tourist destination competitiveness using cognitive mapping and MCDA. Technological Forecasting & Social Change, 131. 2018:147-58. doi:10.1016/j.techfore.2018.01.015.

46. Huang J-H, Peng K-H. Fuzzy Rasch model in TOPSIS: A new approach for generating fuzzy numbers to assess the competitiveness of the tourism industries in Asian countries. Tourism Management. 2012;33:456-65.

47. Zhou Y, Maumbe K, Deng J, Selin SW. Resource-based destination competitiveness evaluation using a hybrid analytic hierarchy process (AHP): The case study of West Virginia. Tourism Management Perspectives. 2015;15:72-80.

48. Assaf GA. Benchmarking the Asia Pacific tourism industry: A Bayesian combination of DEA and stochastic frontier. Tourism Management. 2012;32:1122-7.

49. Hudson S, Ritchie B, Timur S. Measuring destination competitiveness: An empirical study of Canadian ski resorts. Tourism and Hospitality Planning & Development. 2004;1(1):79-94.

50. Enright MJ, Newton J. Determinants of tourism destination competitiveness in Asia Pacific: Comprehensiveness and universality. Journal of Travel Research. 2005;43(4):339-50.

51. Hong WC. Global competitiveness measurement for the tourism sector. Current Issues in Tourism. 2009;12(2):105-32.

52. Zehrer A, Smeral E, Hallmann K. Destination competitiveness—A comparison of subjective and objective indicators for winter sports areas. Journal of Travel Research. 2017;56(1):55-66.

53. Gursoy D, Baloglu S, Chi CG. Destination Competitiveness of Middle Eastern Countries: An Examination of Relative Positioning. Anatolia. 2009;20(1):151-63.

54. Barros CP, Botti L, Peypoch N, Robinot E, Solonandrasana B, Assaf GA. Performance of French destinations: Tourism attraction perspectives. Tourism Managment. 2011;32:141–6.

55. Mendola D, Volo S. Building composite indicators in tourism studies: Measurements and applications in tourism destination competitiveness. Tourism Management. 2017;59:541-53.

56. Assaker G, Hallak R, Esposito Vinzi V, O'Connor P. An empirical operationalization of countries' destination competitiveness using Partial Least Squares Modelling. Journal of Travel Research. 2014;53(1):26-43.

57. Gomezelj DO, Mihalic T. Destination competitiveness—Applying different models, the case of Slovenia. Tourism Management. 2008;29:294-307.

58. JamaicaTouristBoard. Annual Travel Statistics, 2015. Kingston, Jamaica: Research & Market Intelligence Unit; 2016.

59. WTTC. Caribbean resilience and recovery: Minimising the impact of the 2017 hurricane season on the Caribbean's tourism sector. London, U.K.: World Travel & Tourism Council; 2018a.

60. UNWTO. Compendium of Tourism Statistics. Data 2008-2012. Madrid, Spain: World Tourism Organization (UNWTO); 2014.

61. UNWTO. Yearbook of Tourism Statistics. Data 2010-2014. Madrid, Spain: World Tourism Organization (UNWTO); 2016.

62. Dimoska T, Trimcev B. Competitiveness strategies for supporting economic development of the touristic destination. Procedia-Social and Behavioral Sciences 44. 2012:279-88.

63. WTTC. Travel & Tourism Economic Impact Caribbean. World Travel & Tourism Council; 2011.

64. WEF. The Travel & Tourism Competitiveness Report 2019. Travel and Tourism at a Tipping Point. Geneva, Switzerland: World Economic Forum; 2019 04/09/2019. 107 p.

65. Cankurt S, Subasi A. Tourism demand modelling and forecasting using data mining techniques in multivariate time series: a case study in Turkey. Turkish Journal of Electrical Engineering & Computer Sciences. 2016;24:3388-404.

66. Mamula M. Modelling and Forecasting international Tourism Demand – Evaluation of forecasting performance. International Journal of Business Administration. 2015;6(3):102-12.

67. Del Chiappa G, Atzeni M, Ghasemi V. Community-based collaborative tourism planning in islands: A cluster analysis in the context of Costa Smeralda. Journal of Destination Marketing & Management. 2018;8:41-8.

68. Del Chiappa G, Lorenzo-Romero C, Gallarza M. Host community perceptions of cruise tourism in a homeport: A cluster analysis. Journal of Destination Marketing & Management. 2018;7:170-81.

69. Hair JFJ, Anderson RE, Tatham RL, Black WC. Multivariate data Analisys. 5th ed. Madrid: Prentice Hall International, Inc.; 1999. 832 p.

70. Johnson DE. Applied Multivariate methods for data analysis. Kansas, USA: Brooks Cole Publishing Company and ITP Company; 1998.

71. WTTC. New League table summary Regional 2017. London. UK: World Travel & Tourism Council; 2018b.

72. WTTC. Country New League table summary 2017. London, UK: World Travel & Tourism Council; 2018c.

Figure 1. Negative and Positive linear regression

Click here to access/download;Figure;1. Figure 1. Negative and positive linear regression.docx

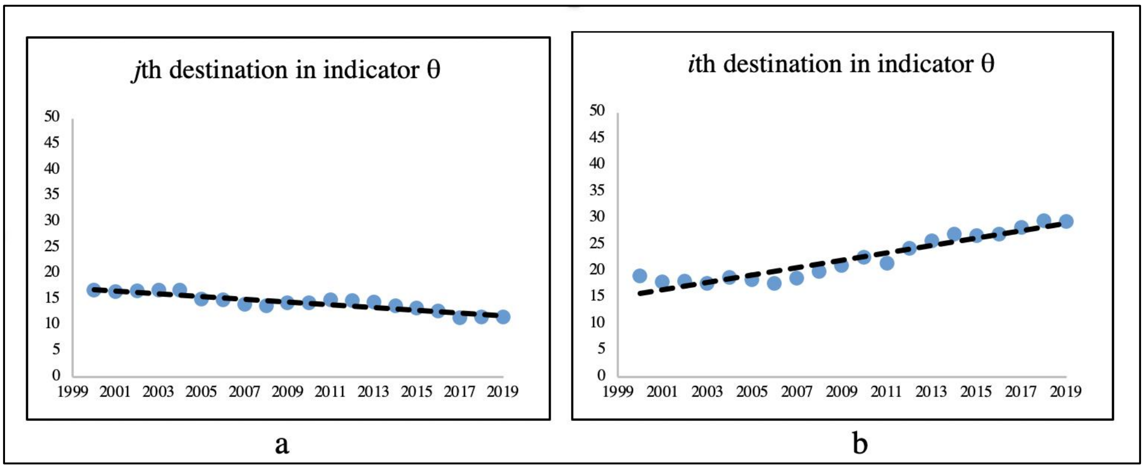

Figure 1. Negative and positive linear regression

Click here to access/download;Figure;3. Figure 2. Clusters.png

Figure 2. Clusters

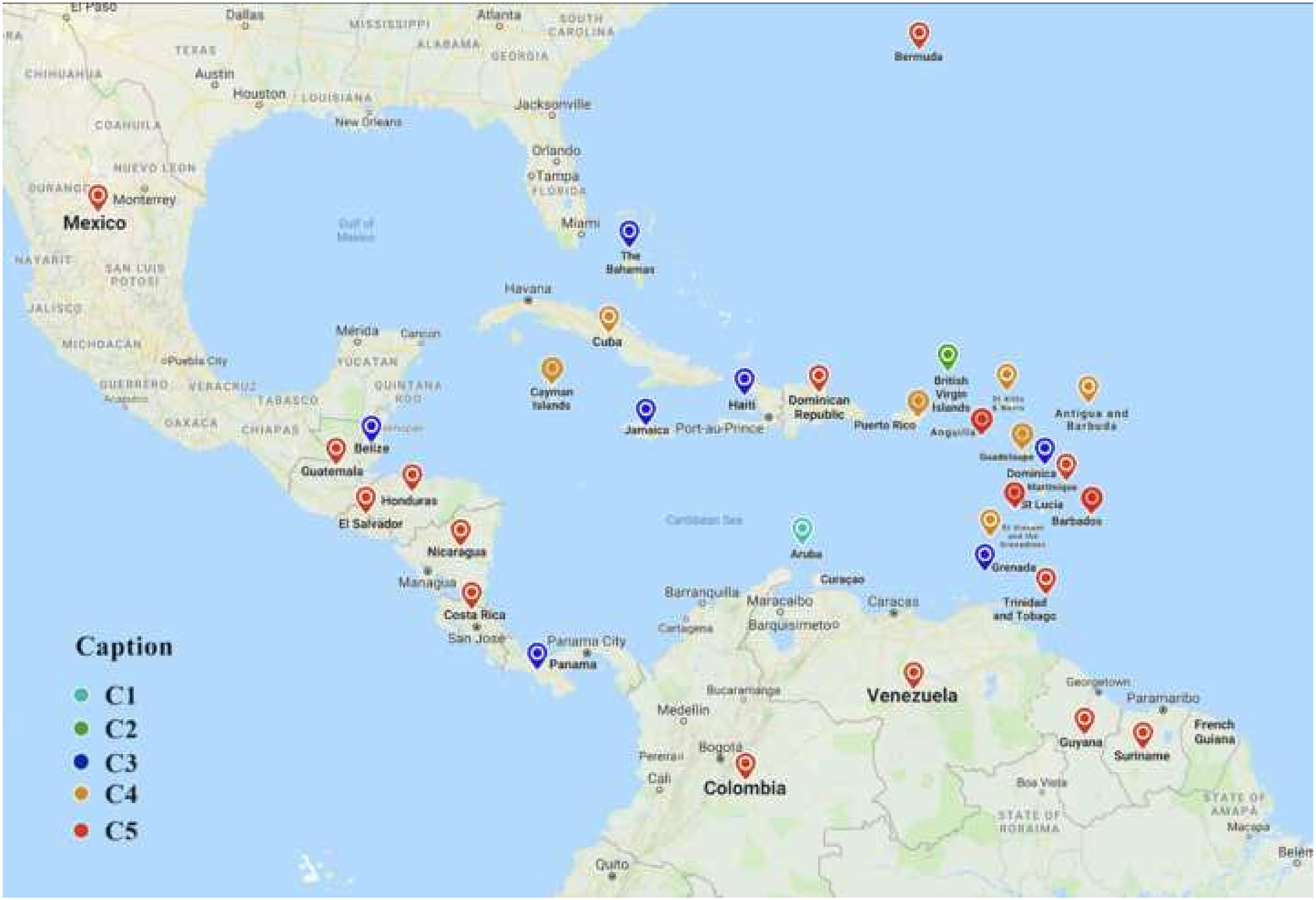

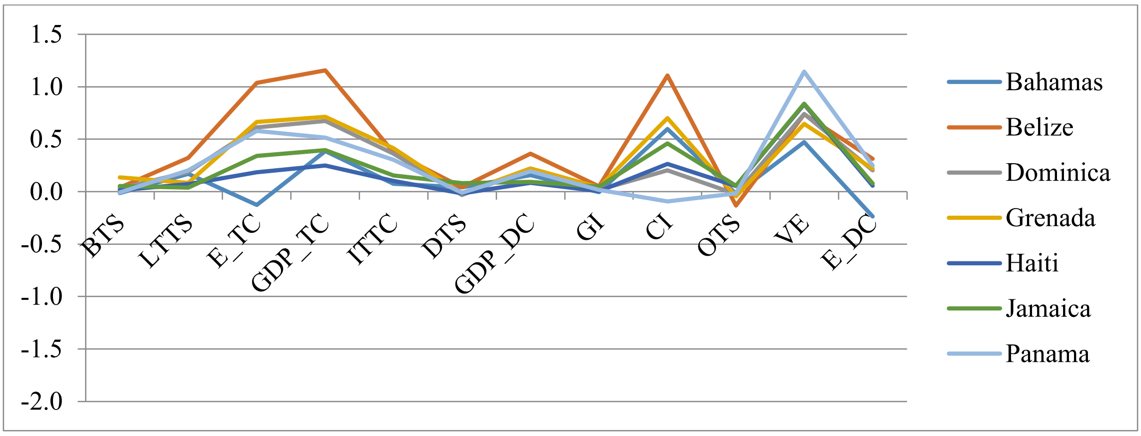

Figure 3. Slopes for the 3rd Cluster

Click here to access/download;Figure;6. Figure 4. Slopes for the 4th cluster.docx

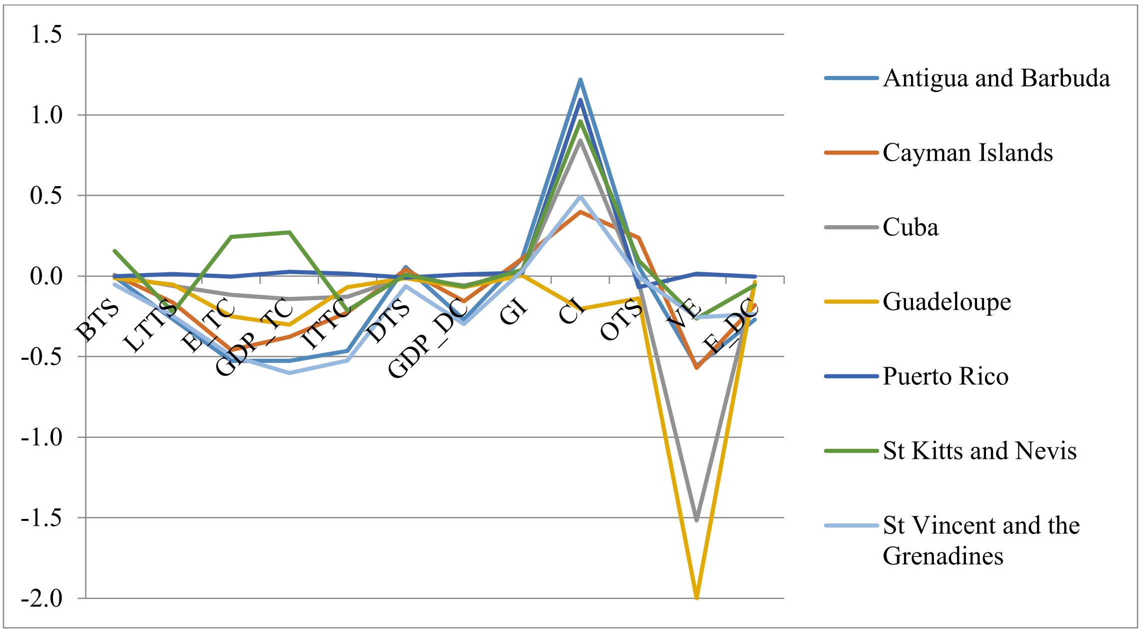

Figure 4. Slopes for 4th cluster

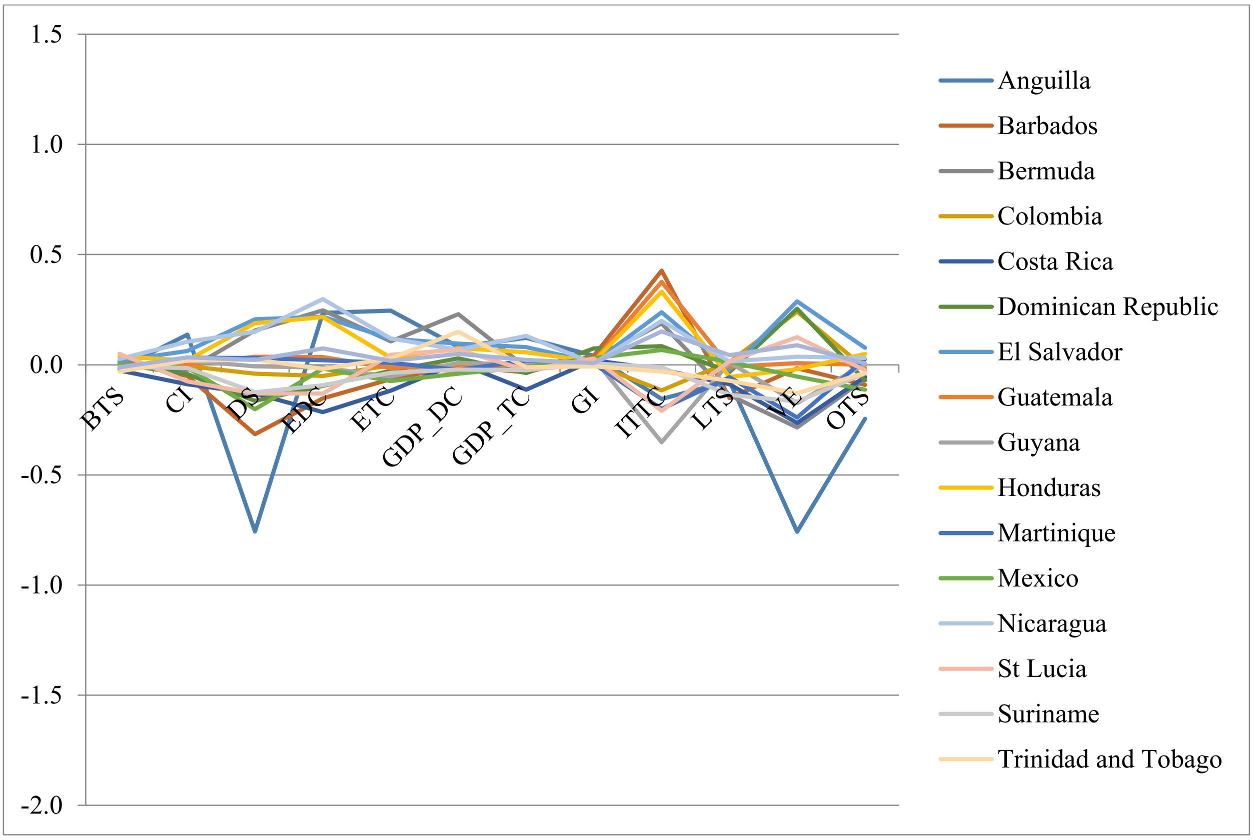

Figure 5. Slopes for 5th cluster

Click here to access/download;Figure;Appendix A.docx

Appendix A.

Figure A.1. Dendrogram

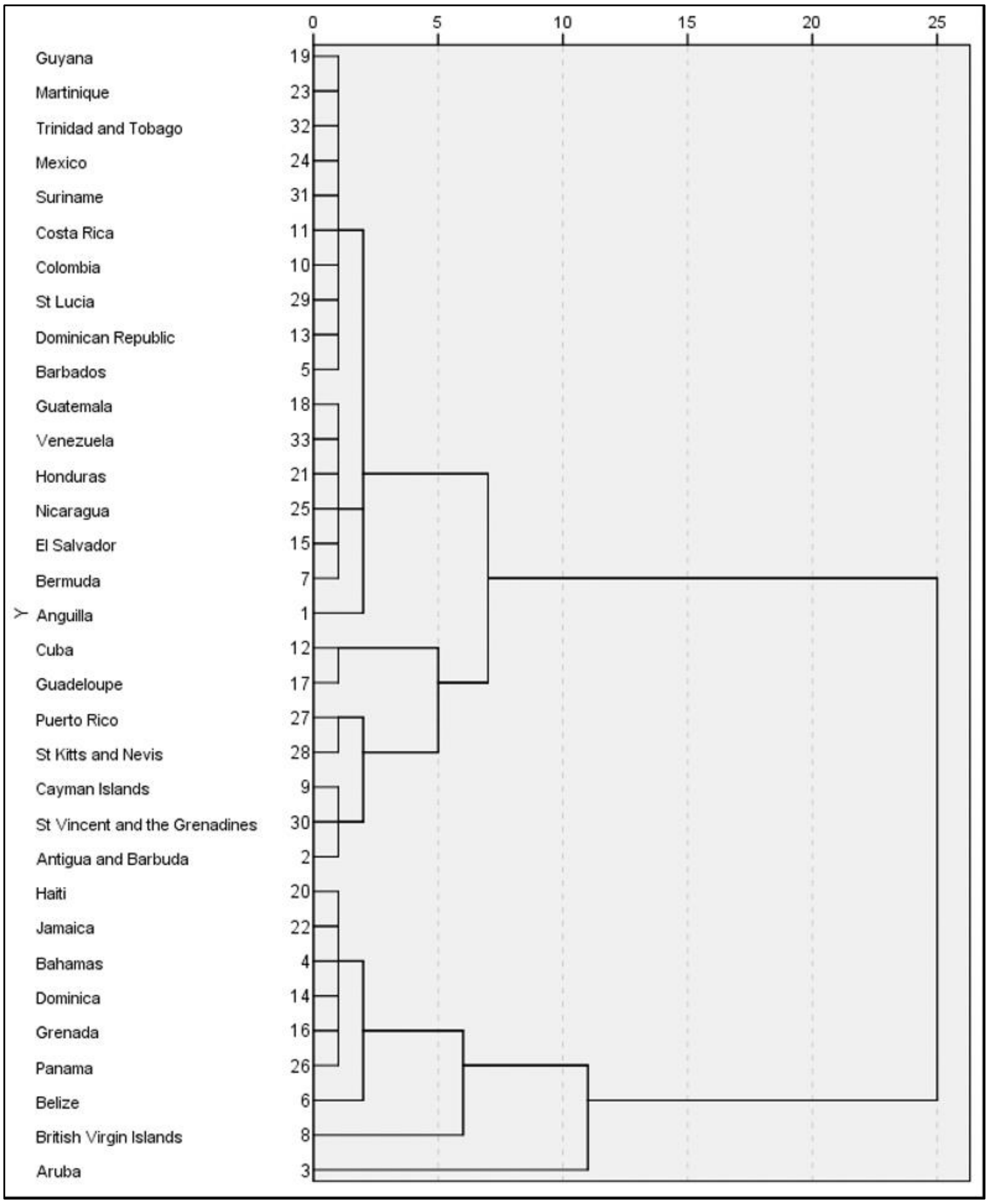

Supplement: Attachment — Submitted filename: PONE-D-20-32669_reviewer comments.pdf [file pone.0252139.s002.pdf]
